# Early Defoliation Techniques Enhance Yield Components, Grape and Wine Composition of cv. Trnjak (*Vitis vinifera* L.) in Dalmatian Hinterland Wine Region

**DOI:** 10.3390/plants10030551

**Published:** 2021-03-15

**Authors:** Ana Mucalo, Irena Budić-Leto, Katarina Lukšić, Edi Maletić, Goran Zdunić

**Affiliations:** 1 Institute for Adriatic Crops and Karst Reclamation, Put Duilova 11, 21000 Split, Croatia; Irena.Budic-Leto@krs.hr (I.B.-L.); Katarina.Luksic@krs.hr (K.L.); Goran.Zdunic@krs.hr (G.Z.); 2 Faculty of Agriculture, University of Zagreb, Svetošimunska Cesta 25, 10000 Zagreb, Croatia; emaletic@agr.hr; 3 Centre of Excellence for Biodiversity and Molecular Plant Breeding, Svetošimunska Cesta 25, 10000 Zagreb, Croatia

**Keywords:** grapevine, leaf removal, cluster thinning, cluster architecture, anthocyanins, proanthocyanins

## Abstract

Defoliation and cluster thinning are of practical importance in a control of the grapevine source-sink balance, cluster architecture, microclimate and berry composition. Nevertheless, their effectiveness on wine composition is unexplored. In this work, the impacts of preflowering (T1), after berry set (T2), and veraison defoliation (T3) and cluster thinning (T4), on yield components, grape and wine composition of cv. Trnjak are given. Implemented techniques significantly reduced yield and affected grape and wine components in comparison to untreated control (C). Despite lowest number of clusters ensured by cluster thinning, defoliation at veraison had lowest yield. Defoliations improved cluster architecture parameters. Highest berry per se was in preflowering T1 and lowest at veraison T3 defoliation. Berries of T1 had lowest sugar content (19.47 °Brix) while T3 had highest (22.3 °Brix), and the reverse is seen in total acidity highest in T1 (6.12 g/L) and lowest in T3 (5.01 g/L). Wines of early defoliations (T1 and T2) had lowest alcohol and highest anthocyanin concentration. Both techniques applied at veraison produced wines with lower anthocyanins and flavonols than those obtained without any intervention (C). In conclusion, the early defoliations (T1 and T2) improve yield and wine composition of cv. Trnjak in the Mediterranean region of Croatia.

## 1. Introduction

Defoliation is one of the promising canopy management techniques to meet the challenges of maintaining the balance between sugar and anthocyanin accumulation through extensive transcriptome rearrangement during ripening [1]. The outcome of this technique is dependent on timing and genotype plasticity [2,3]. Grapevine shoots have pronounced leaf polymorphism; older leaves with larger surface are located along the base and younger, smaller leaves at the top of the shoot. Photosynthetic activity of basal leaves is highest at berry set and decreases afterwards, while photosynthetic activity of apical leaves is the highest during ripening. Photosynthetic activity beside leaf age varies depending on the surface achieved, which can overcome lower photosynthetic rate [4]. Benefits of defoliation are rejuvenation of the canopy form, improvement of cluster microclimate [1], lower yield and looser clusters with smaller berries [4,5]. The stage of development at which berries are exposed to defoliation stress is extremely important as this technique directly affects the disturbance in carbon flux between primary and secondary pathways and indirectly increases sunlight and temperature berry exposure. Moreover, early defoliation stress redirects the vine to the use of nutrient reserves from the trunk and roots [2]. Early timing of basal defoliation versus defoliation at veraison will have a stronger impact on the source sink balance and light and temperature exposure [1].

Increase in grape flavonol and anthocyanin concentration as an outcome of defoliation is due to sunlight driven upregulation biosynthesis of polyphenols [6]. Increase in sun exposure leads to temperature elevation and more pronounced fluctuations in daytime berry temperature in fully exposed clusters [7]. Susceptibility of berries differs depending on the physiological phase of the berry. High temperatures during flowering and fruit set impact berry size, through cell enlargement [8]. Duration of exposure to an interactive effect of temperature, sunlight and water deficit is very important and is still insufficiently explored. Prolonged exposure of Cabernet Sauvignon clusters to direct sunlight initially leads to a decrease in total acidity and increase in sugar, anthocyanins and total phenols until cluster exposure exceeds 100 μmol/m^2^s^1^ of photosynthetically active radiation and decreases afterwards [7]. Research to date has focused on responses of grape berries to two timings of defoliation, before flowering and in veraison, in Sangiovese [1]. The study of preflowering defoliation from four different terroirs in Italy [3] sheds light on the stronger effect of environment than defoliation treatment on transcriptome rearrangement during ripening of Sangiovese. Two different preflowering defoliation intensities in Cabernet Sauvignon revealed a positive impact of 6 leaf removal in contrast to 3 through decreases in yield and average cluster weight and an increase in total phenols, total and individual anthocyanins in wine, mainly malvidin-3-*O*-glucoside [5].

However, negative effects of intense preflowering defoliation of 6 leaf removal are reduction in shoot length, leaf area, trimming and pruning weight and decrease in bud fruitfulness in the following season under restrictive climate conditions [9]. Inconsistencies in studies of basic must and wine composition have been found. Although preflowering defoliation had a tremendous positive impact on color intensity, fruity sensory wine attributes and mouth structure of Pinot Noir wines, an impact on Merlot wines was less effective, while Gamay wines showed no effect at all [2]. Defoliation before flowering and after berry set increased wine color density and total phenol content, with little change in alcohol concentration and pH in Graciano and Carignan wine [10]. Defoliation after berry set had no effect on basic white Malvasia wine composition but increased concentration of hydroxycinammic without consistent effect on hydroxybenzoic acids [11].

In each study there was a maintenance of lateral shoots in cluster zone except in one conducted under temperate climate conditions of Switzerland [2]. It is known that laterals have highest photosynthetic activity during ripening [12] and can cause carry off effect; therefore, those were removed in this study.

The technique of cluster thinning as it results in a significant reduction in yield and high labor costs has been somewhat abandoned except in the table grape production [13], to obtain a better or faster maturation [14] and high quality wine production in cool climate conditions [15]. Impact of this technique on wine is scarcely studied. The most comprehensive work performed during four different vintages [15], revealed the greatest impression in wines of one vintage produced by cluster thinning performed in veraison. However, one weak point is an unclear distinction of cluster thinning magnitude effect from parallel oenological experiment (different yeasts and enzymes) that have surpassed cluster thinning effect in other vintages.

Difficulty in maintaining balanced wines with lower alcohol content and high color intensity in wineries with variety-specific response to treatment applied, as a negative effect of juice substitution treatments on color and polyphenolic profile, in the case of Shiraz [16], underscores the importance of understanding of modulation of polyphenolic compounds at vineyard level.

*Vitis vinifera* L. cv. Trnjak is an ancient red grape variety of very vigorous growth, average yield, with blue berries of soft skin susceptible to *Botrytis cinerea.* It is distributed within an area of protected designation of origin (PDO) in Dalmatian hinterland (Vrgorac, Imotski and Makarska) in Croatia, and the neighboring region of Bosnia and Hercegovina. Trnjak has unique microsatellite profile [17]. There are very sparse viticultural and enology data for Trnjak. Only one research paper reported high amount of total anthocyanins and low amount of low molecular weight flavan constituents, catechin monomers and procyanidin B dimers in wines of Trnjak when compared to Plavac Mali, Merlot, Vranac and Gamay [18]. Full extract, ruby red wines of Trnjak often reach high alcohol rates with residual sugar leading to a physiochemical instability of these wines. Controversially, viticulturists prefer defoliation in veraison despite the occurrence of sunburn damages slightly before veraison and alcohol content of produced wines. To date no detailed research is available for application of canopy manipulation techniques in average yield varieties such as Trnjak.

In this paper, for the first time, the effect of different timings of defoliation (preflowering, after berry set, at veraison) and cluster thinning (at veraison) in terms of agronomic parameters, cluster architecture of grape, ripening parameters of the must and physiochemical and individual polyphenolic profile of produced wines is measured through technological experiment within the same vineyard.

## 2. Results

All treatments significantly affected yield components and grape composition (Table 1), except the number of clusters and pH (in the case of T2). The highest decrease in yield per vine was in veraison defoliation treatment T3 and the lowest in T1. The highest reduction in-between generally similar cluster architecture parameters in defoliation treatments was in T3 and T1, with the exception of the highest average single berry mass in T1.

Early defoliation T1 and T2 treatments lead to a medium compact clusters (class 5), contrary to T3 and T4 treatments in veraison and control that had compact (class 7) and very compact (class 9) clusters, respectively. Regardless of treatments, firmness of berry flesh was soft, and no *Botrytis cinerea* incidence, OIV 459, was detected in any treatment except control characterized as 5. Dried berries were detected in 5 out of 10 clusters in the case of T3 treatment and those were 3, 10, 20, 22 and 30 berries per each cluster. These were not used in physiochemical analysis of ripening parameters but in this case expressed entrance of T3 clusters in over-ripeness and the heterogeneity of berries.

T3 treatment had the highest sugar content (22.30 °Brix) and pH (3.75) and lowest total acidity (5.01 g/L expressed as tartaric acid) opposite to T1 defoliation treatment. Defoliation in T2 and cluster thinning both led to a higher sugar and lower total acidity compared to the control treatment.

According to standard physiochemical parameters (Table 2), wines of traditional time of defoliation in veraison (T3) stand out with statistically greater alcohol (13.9% *v/v*), total dry extract (33.67 g/L) and reducing sugars (2.53 g/L). Although total acidity (6.3 g/L) was also the greatest in these wines, there was no significant difference to those obtained by T2 or T4. Early defoliation treatment was associated with significantly lower alcohol (11.9% *v/v*), reducing sugars (1.67 g/L), total (5.5 g/L) and volatile acidity (0.43 g/L) in comparison to T3. Wines of treatment T1 according to these parameters were close to wines obtained by control treatment C.

Effects of fruit zone leaf and cluster removal on the concentration of nonflavonoid compounds: hydroxycinnamic acids, hydroxybenzoic acids and stilbenes in Trnjak (Table 3) indicate a possibility of natural modulation of those compounds in wine by time adjustment of treatment application. Wines of T3 treatment differed significantly from all others in each nonflavonoid compound, except viniferin, concentration of which was stable in range of 0.19 mg/L in C to 0.22 mg/L in T4. The concentration of total hydroxycnnamic acids was in range 39.42 mg/L in T3 to 49.7 mg/L in T1. However, the lowest values of total hydroxybenzoic acids were 20.92 and 21.53 mg/L determined in treatments T2, and T1 respectively, and the highest 46.58 in T3. The lowest concentration of total stilbenes was determined in traditional treatment T3 and the highest in T4 but without significant difference of this treatment to T1 and T2. Composition of T3 wines differed significantly in predominance of six phenolic acids (caffeic, p-coumaric gallic, protocatechic, vanillic and syringic acid) and resveratrol and lowest concentration of caftaric acid and resveratrol-3-*O*-glucoside.

Defoliation and cluster thinning effect on Trnjak wine flavonoid concentration (Table 4) shows predominance of anthocyanins, specifically, malvidin-3-glucoside followed by acylated and coumarylated forms of malvidin, regardless of treatment. Both early defoliation treatments led to wines rich in anthocyanins, with significant difference between those two only in lower concentration of peonidin-3-(6″acetyl) glucoside in T1. However, wines produced from grapes defoliated in veraison T3 had the lowest concentration of total anthocyanins (82.69 mg/L) and each individual anthocyanin except delphinidin-3-glucoside, delphinidin-3-(6″caffeoyl) glucoside and cyanidin-3-(6″acetyl) glucoside. Significant differences between wines obtained in T3 and T4 treatments in total anthocyanins, and of each individual anthocyanin except delphinidin-3-(6″caffeoyl) glucoside highlight a cluster thinning treatment as better than defoliation in time of veraison. Higher concentration of total and each anthocyanin (except delphinidin-3-glucoside, peonidin-3-glucoside and malvidin-3-(6″-p-coumaroyl) glucoside) in C in comparison to treatments in veraison (T3 and T4) calls into question the cost-effectiveness of those interventions.

Wines of preflowering defoliation had the greatest concentration of total flavonols (35.03 mg/L) and four of eight detected individual flavonol compounds as follows myricetin-3-glucuronide (7.09 mg/L), quercetin-3-glucoside (13.68 mg/L), quercetin-3-galactoside (1.49 mg/L), kaempferol-3-glucoside (2.06 mg/L). There was no significantly lower concentration in other four individual flavonols detected between those and wines obtained from T2, T3, T4 and C. Defoliation at veraison gave wines with the lowest concentration of total flavonols (16.08 mg/L) and of each individual flavonol compounds except 3-glucoside forms of kaempferol, isorhamnetin and syringetin. Wines of canopy manipulation treatments in veraison did not differ from control wines in quercetin-3-glucoside, quercetin-3-galactoside, kaempferol-3-glucoside. Moreover, cluster thinning T4 treatment wines did not differ from the control wines in total flavonol concentration.

Wines of T1 preflowering treatment had the greatest concentration of total (catechin and epicatechin) (32.97 mg/L), catechin (19.08 mg/L), epicatechingallate (8.09 mg/L) and total (43.66 mg/L) and individual B1 (24.04 mg/L) and B3 (4.55 mg/L) dimers. Wines of T2 treatment had the highest concentration of epigallocatechin-gallate, gallocatechin and epigallocatechin and lowest concentration of epicatechin, total (catechin and epicatechin), B2 and B3 dimers and total dimers. The greatest concentration of epicatechin, dimer B2 and B4 was in wines of traditional defoliation T3, while those wines also had the lowest concentration of epicatechingallate.

## 3. Discussion

The preliminary results of the current study shows that defoliation timing influences agronomic, cluster architecture (except EI), ripening parameters of Trnjak grapes and physiochemical (except pH) and polyphenolic composition of wines in comparison to cluster thinning (T4) in veraison and nondefoliated control (C).

Although all treatments had a significant impact on yield loss, this in early defoliation treatments was expected through a decrease in berry set and reduction in cluster weight and compactness [4]. The yield loss recorded at veraison defoliation treatment even exceeds the loss caused by discarding a 35% portion of the grapes using cluster thinning at the same phenological stage. This is a reflection of decrease in cluster and berry weight as a consequence of greater dehydration and sunburn to susceptible berries after sudden exposure to sunlight and temperature increase [1].

Defoliation at a young stage of shoot when the canopy transits from sink to source function leads to a 75% decrease in net CO_2_ exchange rate. Shortly after this is promptly renewed, so defoliated vines reach values the same as control in veraison [12,19]. Although, differences in a bunch weight and number of berries at preflowering defoliation could be explained through delay in the initial growth of berries as in the case of Cabernet Sauvignon under Mediterranean climate [20]. No differences were detected in total and single berry mass between berries of early defoliation treatment in comparison to cluster thinning and control treatment, despite a generally lower number of berries, emphasizing the importance of strong renewal of CO_2_ exchange rate around berry set that overcomes the effect of defoliation [19].

Despite soft firmness of flesh regardless of treatment (OIV 235), no *Botrytis cinerea* incidence (OIV 459) in any treatment except control confirmed the beneficial impact of those techniques on unfavorable microclimate conditions for fungal development [2]. Changes in cluster compactness [21] and berry structure, smaller berries and thicker skin could also be the reason for improvemed resistance of berries to fungal attack [1,2].

T1 defoliation led to the lowest sugar accumulation and pH, and the highest total acidity contraryto previous results conducted on Carignan [10] and Sangiovese of 2 °Brix sugar differential increase after preflowering defoliation in comparison to control and verasion defoliation treatment [1,4]. Decrease in sugar is not accompanied by total acidity as a consequence of simple dilution, which highlights prolongation in initial growth of berries during which sunlight and drought favorited biosynthesis of tartaric acid [22] and higher metabolic consumption rate of sugars occurs [23]. More specifically, early defoliation probably leads to prolongation of lag phase of sugar accumulation that is crucial in reaching required threshold (11.4 °Brix) after which synchronization with anthocyanins starts [24]. Discrepancies between sugar and anthocyanin accumulation early (T1, T2) versus veraison (T3) defoliations makes the timing of defoliation stress an important trigger from primary to secondary metabolite synthesis pathway.

Defoliation at veraison led to the highest sugar content and pH and lowest total acidity, being the reason for the frequent adoption of this technique by viticulturists as in the case of Plavac Mali under Mediterranean conditions of Croatia [25]. The high, 2.83 °Brix TSS accumulation differential between T1 and T3, and consequently the tendency of alcohol decrease for 2% *v/v* by early timing of defoliation underlines this technique as beneficial in light of lower alcohol wine production under Mediteranean climate conditions.

Interestingly, defoliation in T2 and cluster thinning both lead to a higher sugar and lower total acidity compared to untreated control despite clear differences between those two treatments in microclimate conditions of the cluster zone. Contrary to the negative trends of postponing defoliation at berry level, an increase in total acidity with constant pH is seen in wine. Environmentally regulated physiological balances in tartaric and malic acid and tartaric and potassium [26] could explain this reversal between T1 and T3. Moreover, defoliation at veraison led to higher rates in berry heterogeneity due to poorer compensatory power thereafter as reported for Graciano [10]. It accelerated ripening and obviously through dehydration masked the actual effect of reduced sugar accumulation rate [1]. It is well known that dehydration favors leakage of compounds from skin and pulp vacuoles [27] where hydroxycinamic acids are stabilized in form of esters of tartaric acid. Predominance of caftaric (T1 and T2) and caffeic acid (T3) is in accordance with faster kinetic of hydrolysis of those compounds in wine from berries that were sunlight exposed [28].

Among 38 identified and quantified phenolic compounds in wines of different treatments, only two viniferin and isorhamnetin-3-glucoside were constant regardless of the treatment. Our experimental results showed that two treatments of early defoliation T1 and T2 positively influenced wine composition; however, there were huge differences in basic physiochemical parameters and phenolic groups among those. Berry set defoliation treatment T2 and cluster thinning T4 had a similar concentration of individual and total hydroxycinamic acids in accordance with a limited impact of sunlight on biosynthesis of those compounds [29] and various trends reported [11,30]. Besides this, wines of T3 had the lowest content of total hydroxycinamic and caftaric acid and the highest of individual and total hydroxybenzoic acids, primary gallic and protocatechic acid that indicates higher extractability of the later ones from seeds [31].

Early defoliation treatments promoted anthocyanins, flavonols and prodelphinidin monomer flavan-3-ol units in wines with a significantly higher concentration of epicatechin and total sum of procyanidin flavan-3-ol monomers and dimers in T1 wines. On the contrary, T3 defoliation had a depressant effect on high quality associated compounds and a promoting effect on total procyanidin monomer units, individual dimers B2, B3, B4 and total dimers.

Significant difference among anthocyanins in T1 and T2 wines were observed only in the case of peonidin-3-6-acetyl glucoside. Among trisubstituted anthocyanins: 3′,5′-dimethylated anthocyanin malvidin-3-glucoside and its acylated and *p*-coumarylated forms in wine dominated regardless of treatment, likely due to their higher chemical stability, except in the case of T3, in which cyanidin-3-(6″acetyl) glucoside was the third most abundant compound in the anthocyanin profile. Moreover, both veraison treatments promoted cyanidin-3-(6″acetyl) glucoside over petunidin-3-glucoside, and this predominance shows the strong impact of the timing of treatment on anthocyanin profile. Previously, a negative impact of early defoliation treatment on cyanidin acil derivative was found in wines of Cabernet Sauvignon from the continental region of Croatia [5]. Specifically, Trnjak anthocyanins wine profile completely lacks cyanidin-3-glucoside regardless of the treatment, which can be attributed to a greater susceptibility of orthopositioned hydroxyl groups of *o*-diphenols to oxidation in early stages of vinification [32]. It is well known that polyphenolics undergo dynamical changes in wine during aging. Decrease in nonflavonoid and flavonoid compounds hydroxycinammic, hydroxybenzoic acids, anthocyanins, flavonols, flavan-3-ols and proanthocyanidins can be due to a complexation copigmentation process, reactions of polymerization or enzymatic and nonenzymatic degradation. The affinity of individual compounds to enter into those reactions depends on their own solubility and chemical structure and wine matrix reactivity [33,34].

Interestingly, the lowest concentration of each individual anthocyanin was not detected in control samples as we have assumed but in T3, with the exception of 3-glucoside of petunidin and delphinidin, caffeoil derivative of delphinidin and acetil derivative of cyanidin. As in the previous studies, late defoliation treatment also led to an increase in total anthocyanins in Pinot Noir, Merlot [2] and Teran wines [25]; this drastic drop could be due to a rapid increase in berry skin temperature [1]. Sudden exposition of berries to sun at veraison leads to inhibition of anthocyanins biosynthesis or even greater degradation of already synthetized ones [35]. Moreover, a decrease in the total phenolic content and antioxidant activity as active metabolic responses of berry tissue in white varieties to the excess of light further accentuated by strong abiotic stress as photo-oxidative sunburn is reported [36].

Differences in the anatomical structure of berry skin and skin cell wall porosity could also partially influence extractability of those compounds [37]. Smaller berries of preflowering defoliation had two times higher skin thickness and polyphenolic concentration in Pinot Noir at harvest [2] and 13% higher skin thickness compared to veraison defoliated berries in Sangiovese [1]. Those differences in skin integrity could be a reason of differences in berry sensitivity depending on the time of defoliation.

Anthocyanin and flavonol biosynthesis is light dependent [6]. In our case, the timing of modulation of sunlight exposure of closed flowers and small green berries that are 2 to 4 mm in diameter enhanced both groups of polyphenolic compounds. Early defoliation treatments differed from one another in total anthocyanin and flavonol concentration, with a maximum of each in T2 and T1, respectively. Significantly higher concentration of flavonols myricetin-3-glucuronide, quercetin-3-glucoside, quercetin-3-galactoside in T1 treatment are in accordance with the initial accumulation peak of those compounds around flowering and decrease toward berry set [38]. Genome-wide expression analysis during berry ripening revealed that defoliation at either stage resulted in a major transcriptome reprogramming, which slightly delayed the onset of ripening. A closer look at individual gene expression profiles identified revealed a positive impact of preflowering defoliation berry quality traits such as sugar and anthocyanin content [3], whereas defoliation at veraison had a detrimental effect, e.g., less anthocyanin and higher incidence of sunburn damage [1]. Therefore, it is more likely that an increase in anthocyanin and flavonol concentrations in wines of early defoliations is linked to an impact of this technique on phenylpropanoid and flavonoid biosynthesis related genes in grapes trough increase in expression and specific channeling of substrate flow [1,3].

Application of defoliation and cluster thinning technique led to wines with a higher content of flavan-3-ol monomers in comparison with the untreated control, and the highest concentration of those compounds in early defoliated wines, T1 and T2. Gallocatechin, the main prodelphinidin monomer unit in Trnjak wines was almost two times higher in early defoliation treatments in comparison with the control and significantly higher than treatments conducted in veraison. Although those chemically stable compounds are less easily manipulated with preflowering and after berry set defoliation [39], we found significant differences between those two treatments in inversely proportional concentrations of EGCG and EC. Although there was a decrease in epicatechin at various maturity stages of Tempranillo seeds after preflowering defoliation [40], we found minimal concentration of this unit in wines of after berry set T2 defoliation treatment. Two early defoliation treatments differed in impact on EC concentration of Merlot wines [39]. Both, EC and EGC, could be released through acid catalyzed cleavage of interflavanic linkages from gallate ester derivate EGC and EGCG [41]. Differences in EGC and EC indicate various susceptibility of those two wine matrices to chemical reactions with acid catalyzed reaction favorited in T1.Furthermore, the lowest concentration of procyanidin (catechin and epicatechin) in T2 wines, and the high concentration of prodelphinidin units (ECG and EGCG) indicate higher extractability of those compounds from skin [42]. Specifically, only preflowering and veraison defoliation led to a significantly higher concentrations of procyanidins, with a predominance of B1 and B3 in wines of T1 treatment, B2, and B4 in T3 treatment. Wine polyphenolic composition depends on berry ripeness [43], ethanol and wine matrix effect in the disorganization of the outer lipid seed layer [42,44]. In our case, among four different treatments, grapes of T2 reached phenolic maturity, the highest rate of anthocyanins and prodelphinidin units in contrast to procyanidin units in wine [41]. While the predominance of procyanidin units and hydroxybenzoic acids, namely gallic in T3 wine, indicates ethanol favored extraction of those compounds from seeds [42]. Moreover, gallic acid is released in wine through hydrolysis breakdown from epicatechin-gallate esters or proanthocyanidins contained mainly in seeds [45]. A few scenarios of increased flavan-3-ol monomer and dimer concentrations in wines of T1 and T3 that include differences in the morphological structure of the seeds and skin, differences in degree of maturity of the seeds and extractability affected by treatments need further research.

## 4. Materials and Methods

### 4.1. Chemicals

All used chemicals were of high-performance liquid chromatography analytical grade. Phenolic standards of delphinidin-3-*O-*glucoside, cyanidin-3-*O*-glucoside, peonidin-3-*O*-glucoside, malvidin-3-*O*-glucoside, epigallocatechin, procyanidin B1, procyanidin B2, rutin (quercetin 3-*O*-rutinoside) and myricetin were purchased from Extrasynthese (Lyon, France). The standard of caftaric, caffeic, *p*-coumaric, vanillic, syringic and gallic acid and *trans*-resveratrol, (–)-epicatechin, (+)-catechin and (–)-epicatechin-gallate were obtained from Sigma-Aldrich (St. Louis, MO, USA). Kaempferol, quercetin and isorhamnetin were obtained from Fluka (Steinheim, Germany) and quercetin-3-*O*-glucoside from Sigma (St. Louis, MO, USA). Acetonitrile was purchased from J. T. Baker (Deventer, Netherlands). Formic acid and orthophosphoric acid (85% w/w) were purchased from Fluka (Buchs, Switzerland) and ethanol from Kemika (Zagreb, Croatia). Milli-Q water was used for the high-performance liquid chromatography.

### 4.2. Vineyard Site

The experiment was carried out during 2019 growing season at the commercial vineyard in Vrgorac Valley (43°10′9.29″ N, 17°23′44.21″ E, 23 asl), Dalmatian hinterland wine region (Croatia). *Vitis vinifera* L. “Trnjak” grafted on 1103 Paulsen rootstocks was planted in 2007, on a fluvisol soil (loamy, calcareous hydromorphic soil developed on alluvial deposits) on karst at a spacing of 0.6 m (within row) × 2.2 m (between rows) in North-South orientation. Vines were head-trained with vertical shoot positioned trellis (VSP) and sustained with three pairs of catching wires positioned 0.2 m, 0.35 m and 0.35 m, respectively above the basal wire positioned at 0.7 m above the ground. Vines were winter pruned to four canes with two buds each. Canopy manipulation techniques applied before the experiment consisted manually performed shoot thinning when shoots were 20 cm long, according modified Einchorn-Lorenz (E-L) standard at phenological stage 15 [46].

#### Climate Conditions at the Experimental Site

**Figure 1:**
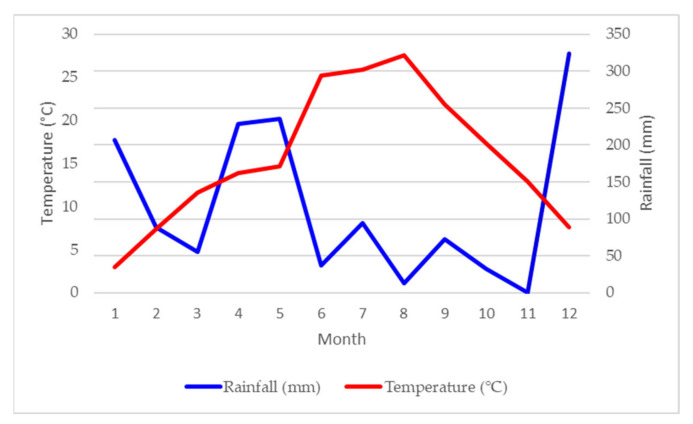
Rainfall (mm) and average temperature (°C) per month of weather station Vrgorac for 2019.

Based on climatic data from the nearest meteorological station located in the city of Vrgorac, the climate in the Vrgorac Valley is considered Mediterranean, with a minimum one-month period of drought in summer. The average annual temperature in 2019 was 15.8 °C, with total annual rainfall of 1389.3 mm. Weather conditions during this experiment at Vrgorac (Figure 1) were very hot and dry. Most of the rainfall was concentrated during winter season (December maximum of 323.7 mm) and early in the growing season (May maximum 235.8 mm). The driest month was August (12.9 mm), which was also a month with the highest average temperature (27.6 °C). In August, the temperature exceeded 30 °C for 3 days in a continuum that can stop or promote the degradation of the color matter. Thermal amplitudes calculated as a difference between daily maximum and minimum temperatures were 10.6, 12.5, 12.3, 13.2 and 12.8 °C from May to August, respectively. Those thermal amplitudes are close to those recorded for the Bordeaux region during ripening period [47]. High thermal amplitudes, with low night temperatures during ripening, can minimize pool depletion of carbohydrate excess for nocturnal growth [48]. Despite those optimal thermal ranges, a detailed dissection of the meteorological data during the period from June to September showed 78 days with a maximal temperature above 30 °C with the highest of all 39.5 °C measured on August 12. For 54 days, the minimum night temperatures were above 20 °C, which can be critical for photosynthesis and respiration balance, color and flavor development.

The Winkler bioclimatic index (WI) is calculated according to the equation from April 1 to October 31 in the Northern Hemisphere [49] for Vrgorac was 2343 °C and is classified as “Region V”.

Winkler bioclimatic index equation:

$$\mathrm{WI}\;=\;\sum_{1\;\mathrm{Apr}}^{31\;\mathrm{Oct}}\left(\mathrm{Tmean}-10℃\right),\;\mathrm{Tmean}\geq 10℃\;\;\;\;\mathrm{Tmean}=\frac{\left(\mathrm{Tmax}+\mathrm{Tmin}\right)}{2}$$

### 4.3. Experimental Set-Up

**Figure 2:**
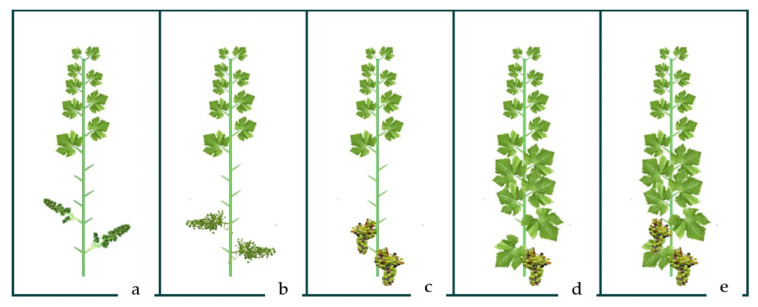
Visual illustration of canopy manipulation techniques of defoliation at preflowering (**a**), berry set (**b**), and veraison (**c**) and cluster thinning in veraison (**d**) in comparison to untreated control (**e**).

A complete randomized block design was used in this experiment to enable three replicates for each treatment. In the middle part of the vineyard, three rows (experimental blocks) were selected with 15 intermediate spacing’s (experimental units) each. Two rows and five intermediated sites were considered as buffer parts of the vineyard with different treatments and were not sampled. Four canopy manipulation treatments (Figure 2) were carried out in specific phenological stages of development according to the E-L standard [46]. Three intense basal defoliation treatments consisted of the manual removal of six leaves from the shoots at different grapevine phenological stages: preflowering (E-L 18), at berry set (bunch at right angle to shoot, berries are 2 to 4 mm diameter) (E-L 27) and at veraison (E-L 35). Green harvest treatment (cluster thinning) was applied at veraison (E-L 35) by cutting 35% of the clusters (yield).

Intervals between first two treatments were nine days, starting on 11 June, while the third treatment was conducted on 6 August 2019.

Control treatment without leaf and cluster removal was also included. The lateral shoots were removed to the proximal node during the berry-set stage as a normal technique in the region. The treatments were randomly assigned to the experimental units across three rows with 15 vines of each treatment per row. Grapes were harvested at the moment of technological maturity of the untreated grapes, manually on September 25.

### 4.4. Yield Components

At harvest, the number of clusters as well as total yield and incidence of *Botrytis cinerea* on each vine per treatment using EPPO guide for visually classification infection symptoms was determined. All the grapes from each treatment were mixed together after weighing to obtain more homogeneous samples for further analysis and winemaking. Immediately after harvest, grapes were transported to the experimental winery with ampelometry of the Institute for Adriatic Crops and Karst Reclamation (Split).

### 4.5. Clusters Characterization

For each treatment, ten representative clusters were randomly selected and cluster structure (weight (g), length (cm), width (cm), number of berries, weight of berries and rachis (g)) were determined. Cluster compactness, firmness of flesh and degree of resistance to Botrytis were evaluated qualitatively using the OIV descriptor N∘ 204, N∘ 235 and N∘ 459 as previously detailed [21].

This descriptor classifies grapevine clusters on five levels, from “very loose” (OIV compactness = 1) to “very compact” (OIV compactness = 9), according to the visibility of the pedicels and the mobility and deformation of the berries.

To obtain quantitative and objective values of compactness, the index CI-12 [50] based on cluster weight and length was calculated according equation:

$$\mathrm{CI}-12=\frac{\mathrm{Cluster}\;\mathrm{weight}\;\left(g\right)}{{\left[\mathrm{Cluster}\;\mathrm{length}\;\left(\mathrm{cm}\right)\right]}^{2}}$$

Cluster elongation (El) was estimated according to equation:

$$\mathrm{CI}=\;\frac{\mathrm{Cluster}\;\mathrm{length}\;\left(\mathrm{cm}\right)}{\mathrm{Cluster}\;\mathrm{width}\;\left(\mathrm{cm}\right)}$$

### 4.6. Analysis of Physiochemical Components of Fresh Juice

Three samples of randomly selected 100 berries per treatment were immediately separated, weighted (g) and processed into juice for rapid physiochemical analysis of basic maturity indicators. Total soluble solids (°Brix) with a portable density meter (RHW-25/°Brix(ATC)), the total acidity content by titration to the point of equivalence with 0.1 M NaOH and the bromthymol blue indicator (expressed as g/L tartaric acid) and pH measured by pH-meter (Methrom 728, Herisau, Switzerland) were analyzed.

### 4.7. Wines

In total 300 kg of grapes, exactly 60 kg per each treatment, were mechanically destemmed, crushed, sulfited to 25 mg/L free sulfur dioxide (SO_2_) and immediately inoculated with commercial yeast strain *Saccharomyces cerevisiae* (Siha 10, Eaton, Langenlonsheim, Germany) at a rate of 250 mg/kg. Homogenized, inoculated pomace of each treatment was divided into three replications, each weighing 20 kg. The pomace cap was mechanically punched down every 12 h during the six days of maceration. After seven days, the pomace was pressed using a small mechanical press and wines were decanted in 20 L glass balloon fermentators on anaerobic completion of fermentation. Once alcoholic fermentation had completely finished, wines were decanted, sulfited to 30 mg/L SO_2_ and stored at 15 °C. Second decanting was performed after three months, SO_2_ corrected to 20 mg/L SO_2_ and wines were bottled in 750 mL bottles. After three months in a bottle, wines were taken for analysis. Microscaled fermentations were performed in experimental winery with ampelometry of the Institute of Adriatic Crops and Karst Reclamation.

### 4.8. Analysis of Standard Components of Wine

Physiochemical enological parameters of the wine were determined according the Official Methods of Wine and Must analysis [51].

### 4.9. Analysis of (Non)Flavonoid Compounds by HPLC

A high-performance liquid chromatography system (HPLC) equipped with diode array (DAD) and fluorescence detector (FLD) (Agilent 1100, Palo Alto, CA, USA) was used for identification and quantification of targeted flavonoid and nonflavonoid phenolic compounds in wines [52]. A priori wine sample preparation included only filtration through a 0.22 μm PTFE membrane filter (Miliford, MA, USA) in a vial of autosampler. The chromatographic separation of the analytes was performed on a Luna Phenyl–Hexyl column (Phenomenex, Torrance, CA, USA) (250 mm × 4.6 mm i.d., 5 μm particle size) with a Phenyl guard column (4.0 × 3.0), tempered at 50 °C. A volume of 20 μL was directly injected into the HPLC system. The gradient solutions used for eluting were two mobile phases: (A) water/phosphoric acid (99.5/0.5, v/v) and (B) acetonitrile/phosphoric acid/water (50/49.5/0.5, v/v/v). The flow rate of the mobile phase was 0.9 mL/min. Detection of phenolic acids was performed on DAD as follows hydroxybenzoic acids at λ = 280 nmx, hydroxycinamates λ = 320 nm, trans-resveratrol at λ = 330 nm. Detection of eluent flavonoid compounds over a nonpolar column of this reverse phased chromatography system was performed on a DAD detector at different wavelengths: flavonols at 360 nm and anthocyanins at 518 nm. Flavan-3-ols were quantitated by a more sensitive fluorescence detector at wavelength excitation λex = 225 nm and wavelength emission λem = 320 nm. Peak identification of the individual flavonoid eluate compounds was performed by comparison of retention times, DAD and the fluorescence spectrum with the external standards [52,53]. The quantitative values of the identified compounds were calculated using the calibration curves and the peak area of the corresponding standard compounds made by external standard analysis and expressed in mg/L.

### 4.10. Statistical Analysis

A one-way analysis of variance (ANOVA) and mean separation using Stats-Fisher’s LSD test (different letters account for significant differences at *p* ≤ 0.05) was conducted to detect differences among the five different treatments. Analysis of variance was conducted in SAS (SAS Institute, Inc., Cary, NC, USA). The results of all physiochemical and flavonoid parameters are expressed as the average ± standard deviation of three replications.

## 5. Conclusions

This study revealed agricultural, cluster architecture, ripening parameters and polyphenolic composition of wines as response to three different defoliation timings and cluster thinning effect in comparison to untreated control. It is worth noting that both early defoliation treatments led to a considerable decrease in alcohol and increase in phenolic content of produced wines. Changes in anthocyanin profile were observed between early defoliation treatments versus both veraison treatments. This powerful impact of timing of treatment application at a single vineyard approach on wine composition is linked to different physiological and metabolic changes at berry level that could not be overcome by environment conditions.

Phenolic ripeness was reached in wines defoliated after berry set, T2 and indicates attained synchronization in ripeness of various parts of berries and coupling the patterns of polyphenolic compounds to that of sugar accumulation. However, traditional defoliation wines of T3 had the highest alcohol content, hydroxybenzoic acids and procyanindins, the main components of seeds, but the poorest content of skin components as total anthocyanins, malvidin-3-glucoside as well as flavonols. Moreover, defoliation at veraison increased the dehydration of berries. Both viticultural interventions at veraison (defoliation T3 or cluster thining T4) were noneffective, as produced wines were lower in anthocyanins and flavonols than those obtained without any intervention (C). According to the presented results, there is no justification for time-consuming cluster thinning as compared to early defoliation treatments. This study provides evidence for the effectiveness of different defoliation timings on the modulation of phenolic composition and the quality improvement of produced wines under Mediterranean climate conditions.

## Figures and Tables

**Table 1:** Agronomic, cluster architecture and ripening parameters of Trnjak grapes subjected to preflowering (T1), after berry set (T2) and veraison (T3) defoliation and cluster thinning (T4) treatments compared to untreated control (C).

	T1	T2	T3	T4	C
*Agronomic parameters*					
Cluster number/vine	11.78 ± 3.67 a	10.36 ± 3.12 b	10.16 ± 2.95 b	7.2 ± 2.46 c	10.67 ± 3.13 ab
Yield/vine (kg)	1.54 ± 0.63 b	1.33 ± 0.52 bc	1.15 ± 0.45 c	1.24 ± 0.51 c	1.83 ± 0.71 a
*Cluster architecture*					
Cluster weight (g)	244.54 ± 34.17 b	251.73 ± 64.22 b	227.47 ± 57.83 b	325.07 ± 54.30 a	360.74 ± 58.93 a
Cluster length (cm)	14.57 ± 2.01 b	15.04 ± 1.04 ab	14.61 ± 1.87 b	15.29 ± 1.28 ab	16.36 ± 1.41 a
Cluster width (cm)	10.49 ± 1.27 ab	10.61 ± 1.26 ab	10.36 ± 2.40 b	12.12 ± 2.19 a	11.77 ± 2.10 ab
Berries/cluster	120 ± 23.63 c	131 ± 33.38 bc	129 ± 39.55 bc	151 ± 28.34 b	183 ± 28.83 a
Rachis weight (g)	6.81 ± 1.14 b	7.43 ± 1.94 b	7.49 ± 2.53 b	10.58 ± 2.69 a	11.53 ± 3.59 a
Total berry weight (g)	237.73 ± 33.37 b	244.30 ± 62.57 b	219.99 ± 55.79 b	314.49 ± 52.41 a	349.21 ± 56.10 a
Mean mass (1 berry)	2.07 ± 0.25 a	1.94 ± 0.29 ab	1.81 ± 0.32 b	2.17 ± 0.25 a	1.98 ± 0.17 ab
Compactness	1.2 ± 0.35 ab	1.11 ± 0.23 b	1.07 ± 0.22 b	1.41 ± 0.32 a	1.36 ± 0.23 a
Cluster elongation	1.43 ± 0.37 a	1.44 ± 0.20 a	1.46 ± 0.29 a	1.29 ± 0.22 a	1.43 ± 0.27 a
*Ripening parameters*					
Weight 100 berries	227.83 ± 3.04 ab	218.84 ± 4.50 cd	214.88 ± 4.89 d	233.85 ± 1.88 a	224.71 ± 4.55 bc
Sugar (°Brix)	19.47 ± 0.15 d	20.37 ± 0.06 b	22.30 ± 0.00 a	20.37 ± 0.12 b	20.10 ± 0.20 c
pH	3.54 ± 0.01 d	3.63 ± 0.01 c	3.75 ± 0.01 a	3.67 ± 0.01 b	3.62 ± 0.02 c
Total acidity (g/L)	6.12 ± 0.05 a	5.30 ± 0.04 d	5.01 ± 0.06 e	5.56 ± 0.09 c	5.87 ± 0.08 b

Results are means of 3 repetitions ± S.D. Means within a row designated by different letters are significantly different by the LSD test at *p* ≤ 0.05.

**Table 2:** Basic physiochemical parameters of Trnjak wines subjected to preflowering (T1), after berry set (T2) and veraison (T3) defoliation and cluster thinning (T4) treatments compared to untreated control (C).

	T1	T2	T3	T4	C
Alcohol (% *v/v*)	11.9 ± 0.0 c	12.5 ± 0.1 b	13.9 ± 0.0 a	12.47 ± 0.4 b	12.1 ± 0.0 c
Total dry extract (g/L)	28.6 ± 0.66 b	28.57 ± 0.29 b	33.67 ± 0.4 a	29 ± 0.17 b	27.8 ± 0.17 c
Reducing sugars (g/L)	1.67 ± 0.06 c	2.2 ± 0.1 b	2.53 ± 0.15 a	1.7 ± 0.2 c	1.87 ± 0.06 c
pH	3.90 ± 0.06 a	3.87 ± 0.02 a	3.89 ± 0.00 a	3.86 ± 0.03 a	3.85 ± 0.02 a
Total acidity (g/L) (as tartaric acid)	5.5 ± 0.44 c	5.97 ± 0.06 ab	6.3 ± 0.00 a	5.93 ± 0.23 ab	5.63 ± 0.06 bc
Volatile acidity (g/L) (as acetic acid)	0.43 ± 0.06 b	0.5 ± 0.0 ab	0.47 ± 0.06 ab	0.47 ± 0.06 ab	0.53 ± 0.06 a

Results are means of 3 repetitions ± S.D. Means within a row designated by different letters are significantly different by the LSD test at *p* ≤ 0.05.

**Table 3:** Effects of fruit zone leaf removal: preflowering (T1), after berry set (T2), at veraison (T3), cluster thinning without defoliation in veraison (T4) and untreated control (C) on the concentration of nonflavonoid phenolic compounds in Trnjak wines (mg/L).

Nonflavonoid Compounds	T1	T2	T3	T4	C
Caftaric acid	46.91 ± 1.5 a	43.02 ± 0.84 b	29.71 ± 2.10 d	43.64 ± 0.91 b	40.29 ± 1.12 c
Caffeic acid	2.78 ± 0.18 c	3.57 ± 0.08 b	8.11 ± 0.27 a	3.32 ± 0.37 b	2.55 ± 0.28 c
*p*-coumaric acid	n.d.	n.d.	1.60 ± 0.08 a	n.d.	n.d.
Total Hydroxycinnamic acids	49.70 ± 1.39 a	46.59 ± 0.92 b	39.42 ± 1.99 d	46.96 ± 1.26 b	42.84 ± 0.90 c
Gallic acid	13.08 ± 0.34 bc	11.47 ± 0.98 c	24.38 ± 1.93 a	13.70 ± 0.3 b	12.09 ± 0.27 bc
Protocatechic acid	2.82 ± 0.19 c	2.87 ± 0.95 c	8.07 ± 0.20 a	4.32 ± 0.20 b	3.66 ± 0.48 bc
Vanillic acid	3.32 ± 0.39 c	3.46 ± 0.21 c	7.00 ± 0.42 a	4.05 ± 0.26 b	4.03 ± 0.16 b
Syringic acid	2.31 ± 0.23 c	3.12 ± 0.12 b	7.13 ± 0.58 a	3.70 ± 0.38 b	3.57 ± 0.29 b
Total Hydroxybenzoic acids	21.53 ± 0.09 c	20.92 ± 0.92 c	46.58 ± 3.10 a	25.78 ± 1.08 b	23.35 ± 0.27 bc
Viniferin	0.21 ± 0.02 a	0.21 ± 0.04 a	0.20 ± 0.05 a	0.22 ± 0.01 a	0.19 ± 0.02 a
Resveratrol-3-*O*-glucoside	2.74 ± 0.09 ab	2.61 ± 0.26 ab	1.78 ± 0.14 c	2.78 ± 0.08 a	2.50 ± 0.06 b
Resveratrol	0.07 ± 0.02 b	0.10 ± 0.03 b	0.16 ± 0.03 a	0.10 ± 0.02 b	0.08 ± 0.01 b
Total stilbenes	3.02 ± 0.11 ab	2.92 ± 0.27 ab	2.14 ± 0.10 c	3.10 ± 0.07 a	2.77 ± 0.08 b

Results are means of 3 repetitions ± S.D. Means within a row designated by different letters are significantly different by the LSD test at *p* ≤ 0.05, n.d.—not detected.

**Table 4:** Effects of fruit zone leaf removal: preflowering (T1), after berry set (T2), at veraison (T3), cluster thinning without defoliation in veraison (T4) and untreated control (C) on the concentration of flavonoid polyphenolic compounds in Trnjak wines (mg/L).

Flavonoid Compounds	T1	T2	T3	T4	K
Delphinidin-3-glucoside	0.72 ± 0.37 a	0.74 ± 0.14 a	0.20 ± 0.17 b	0.68 ± 0.09 a	0.14 ± 0.03 b
Petunidin-3-glucoside	4.01 ± 0.38 a	3.6 ± 0.68 ab	1.42 ± 0.20 d	2.87 ± 0.75 bc	2.34 ± 0.36 cd
Peonidin-3-glucoside	1.09 ± 0.32 a	1.37 ± 0.16 a	0.31 ± 0.04 c	1.33 ± 0.11 a	0.68 ± 0.20 b
Malvidin-3-glucoside	110.27 ± 6.25 a	113.63 ± 2.98 a	61.36 ± 21.45 c	86.59 ± 3.67 b	95.84 ± 6.56 ab
Cyanidin-3-(6″acetyl) glucoside	n.d.	n.d.	2.81 ± 0.3 a	1.95 ± 0.18 b	n.d.
Delphinidin-3-(6″caffeoyl) glucoside	0.36 ± 0.04 a	0.32 ± 0.04 a	0.12 ± 0.03 b	0.10 ± 0.08 b	0.27 ± 0.08 a
Peonidin-3-(6″acetyl) glucoside	1.8 ± 0.12 c	2.74 ± 0.12 a	1.03 ± 0.23 d	2.37 ± 0.20 b	2.1 ± 0.13 b
Malvidin-3-(6″acetyl) glucoside	30.79 ± 6.10 a	32.21 ± 2.82 a	12.97 ± 2.25 c	23.48 ± 2.53 b	28.33 ± 2.31 ab
Peonidin-3-(6″-p-coumaroyl) glucoside	1.28 ± 0.10 a	1.19 ± 0.12 a	0.47 ± 0.11 c	0.74 ± 0.13 b	0.80 ± 0.15 b
Malvidin-3-(6″-p-coumaroyl) glucoside	6.74 ± 1.93 a	6.40 ± 2.7 a	2.0 ± 0.8 b	6.93 ± 1.31 a	6.79 ± 0.68 a
Total anthocyanins	157.05 ± 14.22 a	162.20 ± 8.55 a	82.69 ± 23.31 c	127.04 ± 4.83 b	137.29 ± 10.06 ab
Myricetin-3-*O*-glucuronide	7.09 ± 0.47 a	4.37 ± 0.52 b	0.35 ± 0.15 e	2.12 ± 0.73 d	3.00 ± 0.25 c
Myricetin-3-*O*-glucoside	1.41 ± 0.02 ab	1.28 ± 0.36 b	0.70 ± 0.25 c	1.77 ± 0.10 a	1.62 ± 0.16 ab
Quercetin-3-*O*-glucoside	13.68 ± 0.67 a	11.82 ± 0.17 b	4.82 ± 0.75 c	5.47 ± 0.50 c	5.43 ± 0.02 c
Quercetin-3-*O*-galactoside	1.49 ± 0.10 a	1.16 ± 0.04 b	0.35 ± 0.18 c	0.45 ± 0.10 c	0.38 ± 0.09 c
Kaempferol-3-glucuronide	0.32 ± 0.02 ab	0.30 ± 0.04 ab	0.21 ± 0.07 b	0.35 ± 0.13 a	0.25 ± 0.06 ab
Kaempferol-3-glucoside	2.06 ± 0.05 a	2.03 ± 0.05 a	1.90 ± 0.01 b	1.89 ± 0.01 b	1.86 ± 0.04 b
Isorhamnetin-3-glucoside	0.14 ± 0.02 a	0.16 ± 0.06 a	0.08 ± 0.01 a	0.12 ± 0.03 a	0.15 ± 0.07 a
Syringetin-3-glucoside	8.85 ± 0.33 a	8.88 ± 0.26 a	7.66 ± 0.76 b	7.02 ± 0.30 bc	6.35 ± 0.13 c
Total favonols	35.03 ± 1.57 a	30 ± 0.59 b	16.08 ± 1.46 d	19.19 ± 1.21 c	19.05 ± 0.03 c
Epigallocatechin-gallate (EGCG)	3.62 ± 0.21 c	6.12 ± 0.08 a	4.58 ± 0.21 b	5.52 ± 0.41 a	3.87 ± 0.99 bc
Epicatechingallate (ECG)	8.09 ± 0.67 a	7.59 ± 0.96 a	3.97 ± 0.25 c	6.94 ± 0.85 ab	6.31 ± 0.17 b
Gallocatechin	102.59 ± 3.58 a	110.84 ± 7.16 a	85.95 ± 5.82 b	73.79 ± 7.22 c	66.04 ± 1.38 c
Epigallocatechin	5.94 ± 0.30 a	5.96 ± 0.82 a	n.d.	5.61 ± 0.40 a	n.d.
Dimer B1	24.04 ± 1.49 a	20.31 ± 1.89 b	19.91 ± 3.01 b	19.98 ± 0.65 b	19.52 ± 0.66 b
Catechin	19.08 ± 1.53 a	16.18 ± 1.88 ab	16.55 ± 2.70 ab	16.85 ± 1.23 ab	15.43 ± 0.47 b
Dimer B3	4.55 ± 0.20 a	3.39 ± 0.34 c	4.18 ± 0.24 ab	3.87 ± 0.08 b	3.39 ± 0.19 c
Dimer B4	2.92 ± 0.08 ab	2.55 ± 0.06 cd	3.03 ± 0.19 a	2.71 ± 0.19 bc	2.45 ± 0.05 d
Dimer B2	12.14 ± 1.14 ab	9.30 ± 1.10 c	13.91 ± 2.26 a	11.49 ± 0.94 bc	10.35 ± 0.35 bc
Epicatechin	13.89 ± 1.42 a	10.30 ± 1.48 c	14.46 ± 2.11 a	13.01 ± 0.53 ab	11.38 ± 0.55 bc
Total (catechin+epicatechin)	32.97 ± 2.92 a	26.49 ± 3.35 b	31.01 ± 4.80 ab	29.86 ± 1.74 ab	26.81 ± 1.00 b
Total dimers B	43.66 ± 2.79 a	35.55 ± 2.65 b	41.04 ± 5.42 ab	38.05 ± 1.11 b	35.70 ± 1.11 b

Results are means of 3 repetitions ± S.D. Means within a row designated by different letters are significantly different by the LSD test at *p* ≤ 0.05, n.d.—not detected.

## Data Availability

Not applicable.
